# LPP3 localizes LPA_6_ signalling to non-contact sites in endothelial cells

**DOI:** 10.1242/jcs.172098

**Published:** 2015-11-01

**Authors:** Hiroshi Yukiura, Kuniyuki Kano, Ryoji Kise, Asuka Inoue, Junken Aoki

**Affiliations:** 1 Graduate School of Pharmaceutical Sciences, Tohoku University, 6-3, Aoba, Aramaki, Aoba-ku, Sendai 980-8578, Japan; 2 PRESTO, Japan Science and Technology Agency, Chiyoda-ku, Tokyo 100-0004, Japan; 3 Japan Agency for Medical Research and Development, Core Research for Evolutional Science and Technology (AMED-CREST), Chiyoda-ku, Tokyo 100-0004, Japan

**Keywords:** LPA receptor, LPP3, Endothelial cell, Lysophosphatidic acid

## Abstract

Lysophosphatidic acid (LPA) is emerging as an angiogenic factor, because knockdown of the enzyme that produces it (autotaxin, also known as ENPP2) and its receptors cause severe developmental vascular defects in both mice and fish. In addition, overexpression of autotaxin in mice causes similar vascular defects, indicating that the extracellular amount of LPA must be tightly regulated. Here, we focused on an LPA-degrading enzyme, lipid phosphate phosphatase 3 (LPP3, also known as PPAP2B), and showed that LPP3 was localized in specific cell–cell contact sites of endothelial cells and suppresses LPA signalling through the LPA_6_ receptor (also known as LPAR6). In HEK293 cells, overexpression of LPP3 dramatically suppressed activation of LPA_6_. In human umbilical vein endothelial cells (HUVECs), LPA induced actin stress fibre formation through LPA_6_, which was substantially upregulated by LPP3 knockdown. LPP3 was localized to cell–cell contact sites and was missing in non-contact sites to which LPA-induced actin stress fibre formation mediated by LPA_6_ was restricted. Interestingly, the expression of LPP3 in HUVECs was dramatically increased after forskolin treatment in a process involving Notch signalling. These results indicate that LPP3 regulates and localizes LPA signalling in endothelial cells, thereby stabilizing vessels through Notch signalling for proper vasculature.

## Abstract (precis)

**Summary:** In endothelial cells, the membrane-bound LPA-degrading enzyme LPP3 is specifically expressed at cell–cell contact sites, thereby localizing the signal evoked by extracellularly produced LPA.

## INTRODUCTION

Lysophosphatidic acid (LPA) regulates a wide variety of cellular processes in vertebrates, including migration, adhesion, proliferation, differentiation and cell death, and thereby influences multiple *in vivo* events ranging from organogenesis to development of cancer (Ishii et al., 2004; Lin et al., 2010). LPA is mainly produced by autotaxin (ATX, also known as ENPP2), an extracellular enzyme that converts lysophospholipids to LPA, and exerts its action through at least six G-protein-coupled receptors (LPA_1_–LPA_6_, also known as LPAR1–LPAR6) specific to LPA (Chun et al., 2010). A major question in this field is how extracellularly produced LPA is controlled. Lipid phosphate phosphatases (LPPs) are key factors controlling LPA metabolism. LPPs are integral membrane enzymes that dephosphorylate lipid phosphates such as sphingosine-1-phosphate (S1P) and LPA (Brindley and Pilquil, 2009; Pyne et al., 2005). In vertebrates, at least three LPP genes (*LPP1*, *LPP2* and *LPP3*; also known as *PPAP2A*, *PPAP2C* and *PPAP2B*, respectively) have been identified (Kai et al., 1997; Roberts et al., 1998). LPA, under the control of these LPPs, has recently been shown to be a key regulator of the development of embryonic vasculature in both mice and zebrafish (Escalante-Alcalde et al., 2003; Panchatcharam et al., 2014; Tanaka et al., 2006; Yukiura et al., 2011).

ATX-knockout mice die at around embryonic day (E)9.5 owing to the defects in blood vessel formation in the yolk sac, allantois and embryos (Tanaka et al., 2006). Similar vascular defects are observed when ATX expression is suppressed in zebrafish embryos (Yukiura et al., 2011). In the zebrafish embryos, ATX knockdown causes retarded elongation (or sprouting) of intersegmental vessels (ISVs). Knockdown of several of the LPA receptors (LPA_1_, LPA_4_ and LPA_6_) also results in similar defects in ISV formation (Yukiura et al., 2011). LPP3-knockout mice failed to form a chorio-allantoic placenta and yolk sac vasculature around E9.5 (Escalante-Alcalde et al., 2003). This phenotype is also reproduced in endothelial-cell-specific LPP3-knockout mice (Panchatcharam et al., 2014). Very recently, we established transgenic mice overexpressing ATX and found that overexpression of ATX in the embryo causes similar vascular defects, as well as vascular defects in retina when ATX is overexpressed conditionally in neonates (Yukiura et al., 2015). Because endothelial cells, but not mural cells, are involved in vascular formation in the embryonic stages, it is assumed that the level of LPA, balanced by ATX and LPP3, affects endothelial cell functions by signalling through LPA receptors in a highly coordinated fashion, thereby regulating blood vessel formation. To understand regulation of LPA signalling, we examined the roles of LPA and LPP3 using human umbilical vein endothelial cells (HUVECs).

## RESULTS AND DISCUSSION

**Fig. 1.**
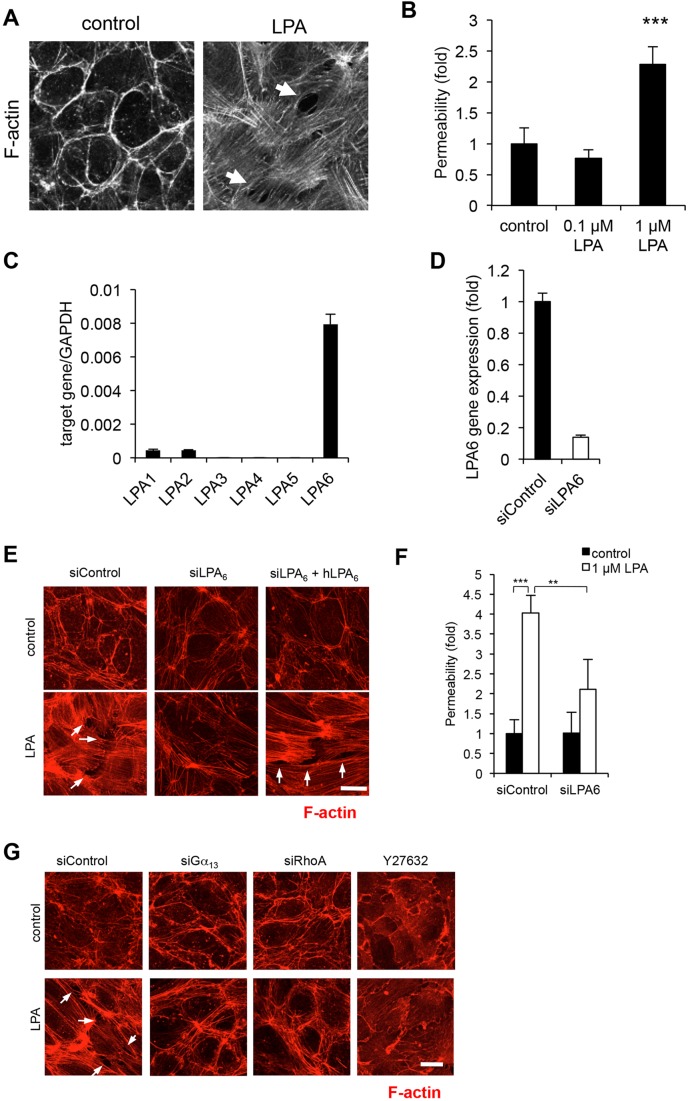
**LPA induces actin stress fibre formation through a LPA_6_–Gα_13_–RhoA–ROCK pathway.** (A) Immunofluorescence analysis of the distribution of F-actin in HUVECs treated with 1 µM LPA as assessed by Alexa-Fluor-594–phalloidin staining. The arrows indicate intercellular gaps. (B) Vascular permeability induced by LPA stimulation as judged by leak of FITC-labeled dextran across the monolayer-cultured HUVECs. Error bars indicate s.d. (*n*=4). ***P<0.001 (one-way ANOVA with Bonferroni's post test analyses). (C) Quantitative (q)RT-PCR analysis of LPA receptors. Total RNA from HUVECs in confluent culture was reverse-transcribed and the resulting cDNAs were subjected to qRT-PCR. The number of the transcripts was normalized to *GAPDH* in the same sample. (D) Gene silencing efficacy of siRNAs for LPA_6_ in HUVECs. 48 h after siRNA treatment, HUVECs were harvested, and LPA_6_ mRNA were quantified by qRT-PCR. siControl, control siRNA. (E) HUVECs were pretreated with siRNA for LPA_6_, or siRNA for LPA_6_ and siRNA-resistant human (h)LPA_6_ cDNA. At 48 h after the transfection, cells were immunostained for F-actin as in A. (F) HUVECs were treated with siRNA for LPA_6_ and LPA-induced vascular permeability was evaluated as in B. Error bars indicate s.d. (*n*=4). **P<0.01, ***P<0.001 (one-way ANOVA with Bonferroni's post test analyses). (G) HUVECs were treated with siRNAs for Gα_13_ and RhoA or with 5 μM Y27632 (ROCK inhibitor). At 48 h after the transfection, cells were immunostained for F-actin as in A. Scale bars: 20 µm.

To examine the role of LPP3 in endothelial cells more precisely, we first examined the effects of LPA on HUVEC functions. We found that LPA dramatically induced the formation of actin stress fibres and intracellular gaps (Fig. 1A), and increased the permeability of the cells (Fig. 1B). We also quantified the LPA effect by counting the number of intercellular gaps (Fig. S1). The most strongly expressed LPA receptor in HUVECs was LPA_6_ (Fig. 1C). The LPA-induced actin stress fibre formation was suppressed by silencing LPA_6_ with small interfering RNA (siLPA6) and restored by reintroducing LPA_6_ in HUVECs (Fig. 1D,E). The LPA-induced increase in the permeability of HUVECs was also suppressed by silencing LPA_6_ (Fig. 1F). LPA-induced actin stress fibre formation and intracellular gaps were suppressed by either treatment with siRNA for Gα_13_ (siGα_13_), siRNA for RhoA (siRhoA), or Y27632, a ROCK inhibitor (Fig. 1G). Consistent with this, we confirmed that activation of RhoA (Fig. S2A) but not Rac1 (data not shown) in LPA-stimulated HUVECs as judged by measuring the level of activated form of RhoA (GTP-bound RhoA).

**Fig. 2.**
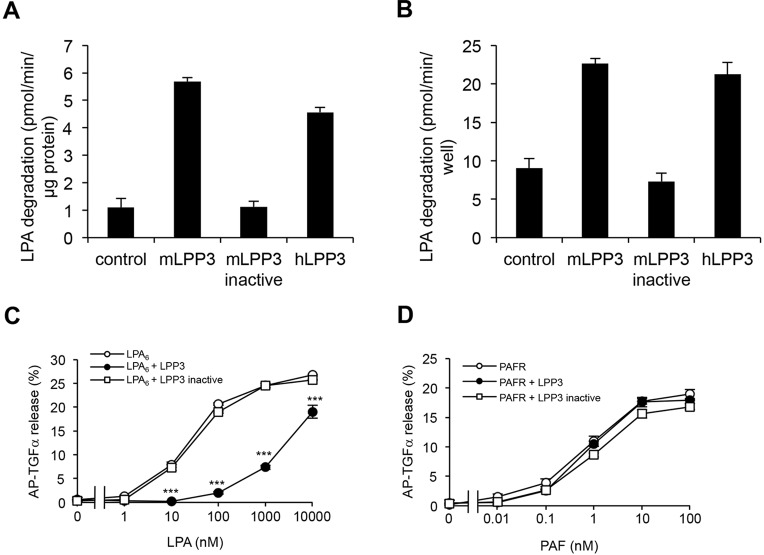
**LPP3 attenuates LPA_6_ activation in HEK293 cells.** The LPA-degrading activity of LPP3 was determined at protein (A) and cellular levels (B). Membrane fractions of HEK293 cells transfected with cDNAs for LPP3 or catalytically inactive LPP3 (A) or the cells themselves (B) were incubated with LPA and the LPP activity was determined by measuring LPA level remaining by LC-MS/MS. Error bars indicate s.d. (*n*=3). mLPP3, mouse LPP3; hLPP3, human LPP3. (C,D) Inhibition of LPA and PAF signalling by LPP3. HEK293 cells that had been transfected with cDNAs for human (h)LPA_6_, hPAFR, hLPP3 and an inactive form of hLPP3 as indicated were stimulated with LPA (C) or PAF (D) at the indicated concentrations, and the activation of receptors was evaluated using TGFα shedding assay. Error bars indicate s.d. (*n*=3). ***P<0.001 (one-way ANOVA with Bonferroni's post-test analyses).

We next characterized LPP3 as an LPA-degrading enzyme. We confirmed that that mammalian LPP3s (mouse and human) degraded LPA both at protein (Fig. 2A) and cellular levels (Fig. 2B). When overexpressed in HEK293 cells, LPP3 prominently suppressed the activation of LPA_6_ but not the activation of the receptor for platelet-activating factor (PAF) in HEK293 cells (Fig. 2C,D) as judged by a transforming growth factor-α (TGFα) shedding assay (Inoue et al., 2012). These results indicate that LPP3 negatively regulates LPA_6_ signalling at the cellular level.

**Fig. 3.**
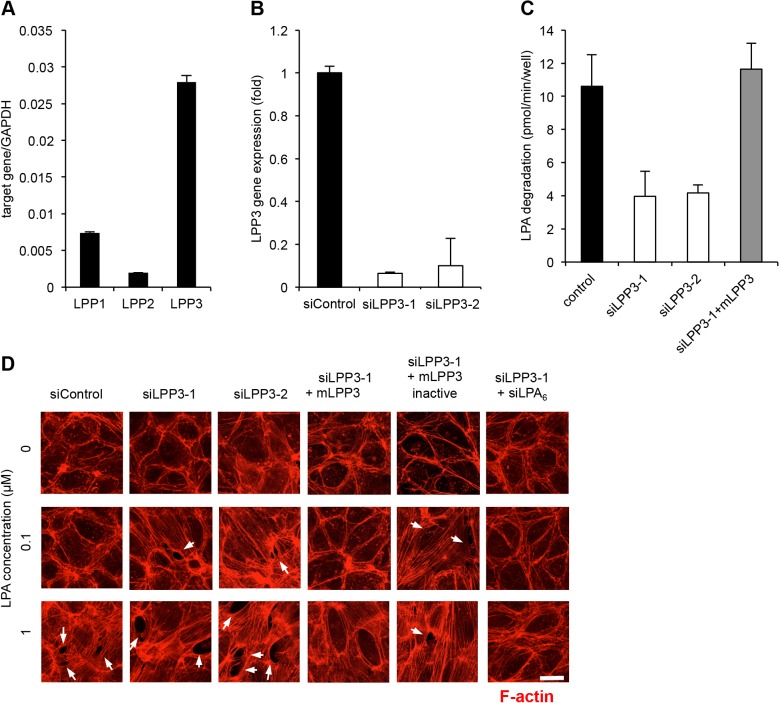
**LPP3 negatively regulates LPA signalling in endothelial cells.** (A) Expression of LPPs in HUVECs. Quantitative (q)RT-PCR was performed as in Fig. 1C. (B) Gene silencing efficacy of two siRNAs for LPP3 in HUVECs as judged by qRT-PCR. (C) LPA-degrading activity in HUVECs. The LPA-degrading activity of HUVECs transfected with siRNAs for LPP3 and/or mLPP3 cDNA in combination was evaluated by measuring the LPA level remaining in the culture medium by LC-MS/MS. (D) Staining of F-actin in HUVECs. HUVECs were transfected in combination with siRNAs for LPP3 and LPA_6_ and mouse LPP3 (mLPP3, both active and inactive form) cDNA as indicated. At 48 h after transfection, HUVECs were stimulated with LPA at the indicated concentration. The arrows indicate intercellular gaps. Scale bar: 20 µm.

We next examined the effect of knockdown of LPP3, which was found to be highly expressed in HUVECs (Fig. 3A). In control HUVECs, treatment with 0.1 µM LPA but not 1 µM LPA failed to induce the rearrangement of the cytoskeleton and the disruption of cell–cell adhesion (Fig. 3D). Knockdown of LPP3 by siRNA treatment (siLPP3) resulted in lower adhesion of HUVECs to cell culture plate (data not shown) and lower LPA-degrading activity in HUVECs (Fig. 3B,C). In the HUVECs treated with siRNA for LPP3, the lower LPA concentration (0.1 µM) did induce actin stress fibre formation and disrupted cell–cell adhesion (Fig. 3D). The effect was reversed by re-expression of mouse LPP3 (mLPP3) but not by catalytically inactive mLPP3, in which the catalytic serine 198 is replaced with threonine (Fig. 3D). The effect of LPP3 knockdown could be attributed to an enhanced LPA_6_ signalling, because the effect of siRNA for LPP3 was negated by simultaneous knockdown of LPA_6_ in the cells (Fig. 3D). These results were confirmed by quantification of the number of LPA-induced intercellular gaps (Fig. S1B). In addition, we confirmed that knockdown of LPP3 by siRNA treatment resulted in enhanced RhoA activation induced by LPA (Fig. S2B).

**Fig. 4.**
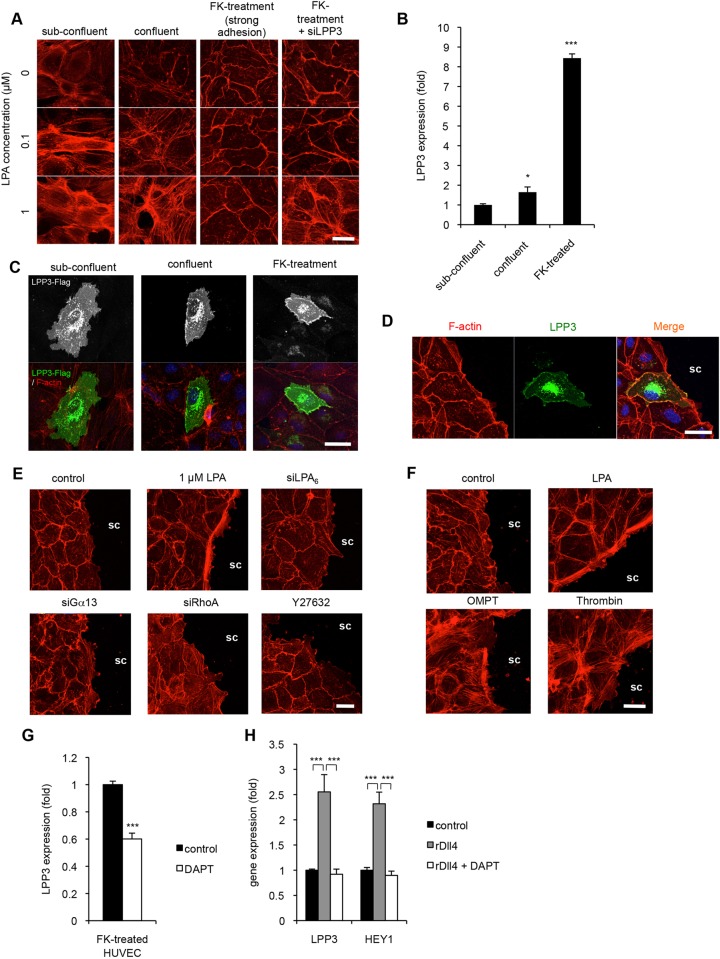
**LPP3 determines the subcellular localization of LPA signalling in endothelial cells.** (A) Sub-confluent, confluent and forskolin (FK)-pretreated (for 30 min) HUVECs were stimulated with LPA at the indicated concentration for 30 min. F-actin was stained with Alexa-Fluor-594–phalloidin (red). Unlike sub-confluent and confluent HUVECs, cells with strong adhesion did not respond to LPA, which was reversed by treatment of the cells with siRNA for LPP3. (B) Cell-density-dependent expression of LPP3 in HUVECs. Expression of LPP3 in sub-confluent, confluent and forskolin-treated (2 h) HUVECs was determined by quantitative (q)RT-PCR. Error bars indicate s.d. (*n*=4). (C,D) Subcellular localization of LPP3. HUVECs were transfected with cDNA for Flag-tagged LPP3. (C) HUVECs (sub-confluent, confluent or forskolin-treated) were stained with anti-Flag antibody (green) and Alexa-Fluor-594–phalloidin (red). (D) Forskolin-treated HUVECs were scratched using a tip, and after 30 min cells were stained as in C. sc, scratched area. (E,F) Leader cells were highly susceptible to LPA stimulation. (E) Confluent HUVECs were scratched, and after 30 min cells were stimulated with 1 µM LPA and stained with Alexa-Fluor-594–phalloidin. HUVECs were also pre-treated with siRNAs for LPA_6_, Gα_13_ or RhoA, or with 5 μM Y27632. (F) The scratched HUVECs were also stimulated with OMPT (a phosphatase-resistant LPA analog, 1 µM) or thrombin (1 U/ml). (G,H) Notch-induced expression of LPP3 in HUVECs. (G) Forskolin-induced LPP3 expression in HUVECs was suppressed by Notch inhibitor, DAPT (10 µM for 2 h). (H) The Notch ligand, recombinant (r)Dll4 (1 µg/ml), enhanced the expression of LPP3 in HUVECs, which were suppressed by DAPT. Another Notch-target gene, HEY1, showed a similar expression pattern. At 24 h after rDll4 stimulation, expression of LPP3 and HEY1 was determined by qRT-PCR. Error bars indicate s.d. (*n*=4). *P<0.05, ***P<0.001 (one-way ANOVA with Bonferroni's post-test analyses in B and H, and Student's *t*-test in D). Scale bars: 20 μm.

During our experiments using HUVECs, we noticed that the effect of LPA was highly affected by cell density, especially by the presence of cell–cell contact. Sparse HUVECs were more susceptible to LPA than confluent HUVECs as shown by the formation of actin stress fibres (Fig. 4A). This might be due to a lack of cell–cell adhesion, because HUVECs treated with forskolin, which enhances cell–cell adhesion (Fukuhara et al., 2005; Stelzner et al., 1989), showed marked resistance to LPA stimulation (Fig. 4A). Interestingly, the effect of forskolin was negated by LPP3 knockdown (Fig. 4A), indicating that LPP3 is involved in the resistance to LPA in forskolin-treated HUVECs. This was confirmed by quantification of the number of cells with actin stress fibres (Fig. S1C), given that evaluation of LPA effect based on intercellular gaps cannot be applied to sub-confluent cells. We also found that, in forskolin-treated cells, LPA-induced RhoA activation was significantly attenuated (Fig. S2C). Furthermore, the LPP3 expression level was ∼1.5 fold higher in confluent HUVECs than in nonconfluent HUVECs, and was dramatically increased in forskolin-treated HUVECs (Fig. 4B). The forskolin-induced upregulation of LPP3 was prominently suppressed by a protein kinase A (PKA) inhibitor (H89) but not by a cAMP-dependent exchange protein (EPAC) inhibitor (ESI-09) (Fig. S3). We also found that in forskolin-treated HUVECs, LPP3 was predominantly localized to cell–cell contact sites (Fig. 4C). Co-staining with anti-VE-cadherin antibody confirmed the result (Fig. S4A). Such subcellular localization of LPP3 was not observed in either confluent or nonconfluent cultures (Fig. 4C). In HUVEC monolayers scratched with tips, LPP3 was not localized to the site where neighbouring HUVECs were absent (non-contact site, Fig. 4D; Fig. S4B). Interestingly, leader cells, that is, cells located at the edge of the HUVEC sheets, showed strong actin stress fibre formation after LPA stimulation (Fig. 4E), indicating that these cells dramatically responded to LPA. These results indicate that the site of LPA action is restricted to the non-contact site of HUVECs. This effect was negated by treating the cells with siRNAs for LPA_6_, Gα_13_ or RhoA, and upon Y27632 treatment (Fig. 4E). The localized LPA_6_ signalling to the non-contact sites appeared to be specific for LPA, because it was not induced by the protease-activated receptor (PAR) ligand thrombin (Fig. 4F). We also applied a phosphatase-resistant LPA_6_ agonist, OMPT (Jiang et al., 2013), and found that OMPT induced formation of actin stress fibres in all HUVECs (Fig. 4F). These results indicate that the localized activation of LPA_6_ at non-contact sites in the leader cells was due to the absence of LPP3 in these sites.

In endothelial cells, contact-dependent gene expression is partly regulated by Notch signalling. The enhanced expression of LPP3 in forskolin-treated HUVECs was suppressed by the Notch inhibitor DAPT (Fig. 4G). Furthermore, the expression of LPP3 was upregulated when HUVECs were cultured on a plate coated with Dll4 (Fig. 4H) but not jagged-1 (data not shown). We also found that on a Dll4-coated plate LPA-induced RhoA activation was significantly attenuated (Fig. S2D). Thus, in endothelial cells, LPA signalling appears to be regulated by Notch signalling through modulation of LPP3 expression.

Recently, LPA has been found to enhance vascular permeability upon inflammation (Panchatcharam et al., 2014). Thus, LPA seems to be a vasculature-destabilizing factor in pathological conditions. In this study, we found that LPP3 enhanced the cell–cell interactions by downregulating LPA_6_ signalling. Previous reports have also indicated that LPP3 enhances cell–cell interaction through its RGD domain (or RGE domain in mice LPP3) and integrins (Humtsoe et al., 2003, 2005; Wary and Humtsoe, 2005). Thus, LPP3 appears to stabilize blood vessels through several mechanisms.

LPA is considered to be ubiquitously distributed because its synthetic enzyme, ATX, and its precursor, lysophosphatidylcholine (LPC), are also ubiquitously distributed (Aoki et al., 2008). In addition, many types of endothelial cells express high levels of LPA_6_ (Ren et al., 2013). These findings appear to indicate that LPA is always active and induces massive vascular permeability. However, as we have shown in this study, this is not the case because LPP3 is highly expressed in endothelial cells, which protects them from the LPA effect. We demonstrate that LPP3 was expressed locally and restricted LPA signalling at the subcellular level. LPA signalling was profoundly suppressed at cell–cell contact sites, where LPP3 was accumulated, and was fully active in non-contact sites, where LPP3 was absent (Fig. 4). Jia et al. have reported that a dityrosine motif present in the second cytoplasmic portion of LPP3 serves as a basolateral targeting signal in MDCK cells (Jia et al., 2003). It is likely that HUVECs use a similar mechanism to locate LPP3 to the cell–cell contact site (lateral site). Interestingly, another LPP isoform, LPP1, distributes specifically to the apical surface of MDCK cells. Thus, it is possible that each LPP isoform regulates lipid phosphate signalling in specific domains of the cells.

We also found that LPP3 expression is regulated transcriptionally by Notch signalling (Fig. 4G,H). Notch signalling is known to contribute to the stability of newly formed vessels by inducing gene expression and establishing firm adherens junctions (Phng and Gerhardt, 2009). Thus, it is reasonable to assume that Notch signalling stabilizes newly formed vessels by inducing LPP3 and downregulating LPA signalling.

## MATERIALS AND METHODS

### Reagents and antibodies

1-Oleoyl LPA was purchased from Avanti Polar Lipids Inc. Stealth siRNAs against human LPP3, LPA_6_, Gα_13_ and RhoA were purchased from Invitrogen. DAPT, thrombin, forskolin, Y27632 and anti-Flag M2 antibody were purchased from Sigma. Alexa-Fluor-594–phalloidin was purchased from Molecular Probes. Recombinant human Dll4 was from R&D Systems.

### Cell culture and transfection

HUVECs were purchased from Kurabo and were maintained in HuMedia-EG2 with a growth additive set. HEK293T cells were maintained in Dulbecco's modified Eagle's medium (DMEM; Nissui, Tokyo, Japan) containing 10% fetal calf serum and antibiotics. HUVECs and HEK293 cells were transfected by using the NEON electroporation system (Invitrogen) and Lipofectamine 2000 (Invitrogen), respectively.

### Permeability assay

The permeability assay was performed as described previously (Fukuhara et al., 2005) using FITC-labelled dextran (molecular mass, 42,000 kDa) as an indicator of permeability.

### Evaluation of G-protein-coupled receptor activation

Activation of LPA_6_ receptor and PAF receptor were evaluated by a TGF-α shedding assay as described previously (Inoue et al., 2012) using cDNAs for human LPA_6_ and human PAFR, except that cDNA for human LPP3 was co-transfected.

### LPA degradation assay

Cells (HUVECs or HEK293 cells transfected with cDNA for LPP3) or a membrane fraction from cells that had been transiently transfected with LPP3 plasmid constructs, were incubated in M199 containing 1% BSA and 10 µM LPA. The amount of LPA remaining in the medium was determined by liquid chromatography tandem mass spectrometry (LC-MS/MS) as previously described (Inoue et al., 2011).

### Immunofluorescence staining

For immunofluorescence cell staining, HUVECs were cultured on collagen-I-coated glass-bottomed dishes, fixed with 4% PFA in PBS, permeabilized in 0.1% Triton X-100 in PBS for 10 min, incubated with 3% BSA in PBS containing 10% goat serum, and incubated with Alexa-Fluor-594-conjugated phalloidin at room temperature. Images were captured and line scan plot analysis was performed with a Zeiss LSM 700 confocal laser scanning microscope. Quantification of the data was performed by counting the number of intercellular gaps or the number of cells with or without actin stress fibre.

### Quantitative RT-PCR analysis

Total RNA was reverse-transcribed using High-Capacity cDNA RT Kits (Applied Biosystems) according to the manufacturer's instructions. PCR reactions were performed with SYBR Premix Ex Taq (Takara Bio) and were monitored by ABI Prism 7300 (Applied Biosystems). Standard plasmids ranging from 10^2^ to 10^6^ copies per well were used to quantify the absolute number of transcripts of cDNA samples. The numbers of transcripts were normalized to the number of a house-keeping gene, *GAPDH* in the same sample.

Primers used in human gene expressions are: LPP1, 5′-CTGGAGCGATGTGTTGACTG-3′ and 5′-GTTGGTGTTTCATGCAGAGTTG-3′; LPP2, 5′-CTACCGTCCAGATACCATCACC-3′ and 5′-GTTGAAGTCCGAGCGAGAATAG-3′; LPP3, 5′-TGGCAGGATTTGCTCAAGG-3′ and 5′-CAATAATGTCCACAGGTGAAAGGG-3′; and HEY1, 5′-GAGAAGCAGGGATCTGCTAA-3′ and 5′-CCCAAACTCCGATAGTCCAT-3′. Note that primer sets for LPA_1_–LPA_6_ and GAPDH were the same as previously described (Inoue et al., 2011).

### Evaluation of activation of RhoA and Rac1

Activation of RhoA and Rac1 was examined using a G-LISA system (Cytoskeleton) according to the manufacturer’s instruction. In this system, the level of activated (GTP-bound) RhoA and Rac1 was determined using antibodies specific to the activated form of RhoA and Rac1.

### Statistical analyses

All statistical analyses were carried out using Prism software (GraphPad). *P<*0.05 was considered to be significant.
